# Supplementary material for: Altered intercellular communication and extracellular matrix signaling as a potential disease mechanism in human hypertrophic cardiomyopathy
Source: Sci Rep. 2022 Mar 25;12:5211. doi: 10.1038/s41598-022-08561-x (PMC8956620; doi:10.1038/s41598-022-08561-x)
Supplement: Supplementary file 1 — Supplementary Information. [file 41598_2022_8561_MOESM1_ESM.pdf]

Supplemental Table ST1. Patient Characteristics.

Patient	1	2	3	4	5	6	7	8	9
Demographics									
age at myectomy	43	55	54	54	73	58	54	63	60
female	no	yes	yes	no	yes	no	no	no	no
nyha class>3	yes	yes	yes	yes	yes	yes	no	yes	yes
Med Hx									
Prior AF	no	no	no	yes	no	no	no	yes	no
Prior VT/VF	no	no	no	no	no	no	no	no	no
Prior NS VT	no	no	no	no	no	no	no	no	no
Prior syncope	no	no	no	no	no	no	yes	no	no
Fam Hx SCD	no	no	yes	no	no	no	no	no	no
Fam Hx HCM	no	yes	yes	no	no	no	no	no	no
Comorbidities	none	CAD, HTN, HLD, COPD, DM2, OSA, Spinal Stenosis	HTN, HLD	HTN	OSA, HTN, CAD, HLD	HTN, HLD, CAD	none	HLD	prostate CA, OSA
Meds									
beta blocker	yes	yes	yes	yes	no	yes	no	yes	yes
calcium channel blocker	no	no	no	yes	yes	no	yes	no	no
ACE or ARB	no	no	no	yes	no	no	no	no	no
Diuretic Use	no	no	no	no	no	yes	no	no	no
loop diuretic	no	no	no	no	no	yes	no	no	no
thiazide	no	no	no	no	no	no	no	no	no
potassium sparing	no	no	no	no	no	no	no	no	no
disopyramide	no	no	no	no	no	no	no	no	no
amiodarone	no	no	no	no	no	no	no	no	no
Physiological measurements									
LA size (mm)	52	46	40	69	49	54	35	57	39
systolic blood pressure	128	110	106	124	126	142	140	126	140
diastolic blood pressure	82	80	60	78	78	90	80	78	80
IVS thickness (mm)	13	15	22	24	15	15	15	18	18
Posterior wall thickness	12	12	13	11	8.9	8.7	9.6	14	13
LVEF (%)	70	65	65	60	65	65-70	65	65	70
LVEDD (mm)	45	36	36	43	31	48	40	44	33
LVESD (m)	29	23	22	33	22	33	24	25	21
SAM	yes	yes	yes	yes	yes	yes	yes	yes	no
MR	moderate	mild	mild	moderate to severe	mild	trace	mild	trace	mild
LVOT gradient rest (mm Hg)	60	35	110	0	0	0	75	100	0
LVOT gradient provocation (mm Hg)	NA	85	NA	40	150	110	NA	NA	90-100
LGE on MRI	none	mild	ND	moderate	none	none	ND	none	mild
Surgical Procedure	extended septal myectomy with mitral valve repair	extended septal myectomy, CABGx1	extended septal myectomy	extended septal myectomy, MV repair, MAZE	extended septal myectomy	septal myectomy and MV repair	extended septal myectomy	extended septal myectomy, MAZE	extended septal myectomy
Pathogenic HCM Variant	NF	NF	NF	KRAS	NF	NF	NF	NF	MYBPC3

Abbreviations: NYHA = New York Heart Association; AF = atrial fibrillation; VT/VF = ventricular tachycardia or ventricular fibrillation; NSVT = nonsustained ventricular tachycardia; SCD = sudden cardiac death; HCM = hypertrophic cardiomyopathy; CAD = coronary artery disease; HTN = hypertension; HLD = hyperlipidemia; COPD = chronic obstructive pulmonary disease; DM2 = diabetes mellitus, type 2; OSA = obstructive sleep apnea; DI = diabetes insipidus; GERD = gastroesophageal reflux disease; AS = aortic stenosis; MR = mitral regurgitation; NF = not found; ND = not done; ICD = implantable cardioverter-defibrillator

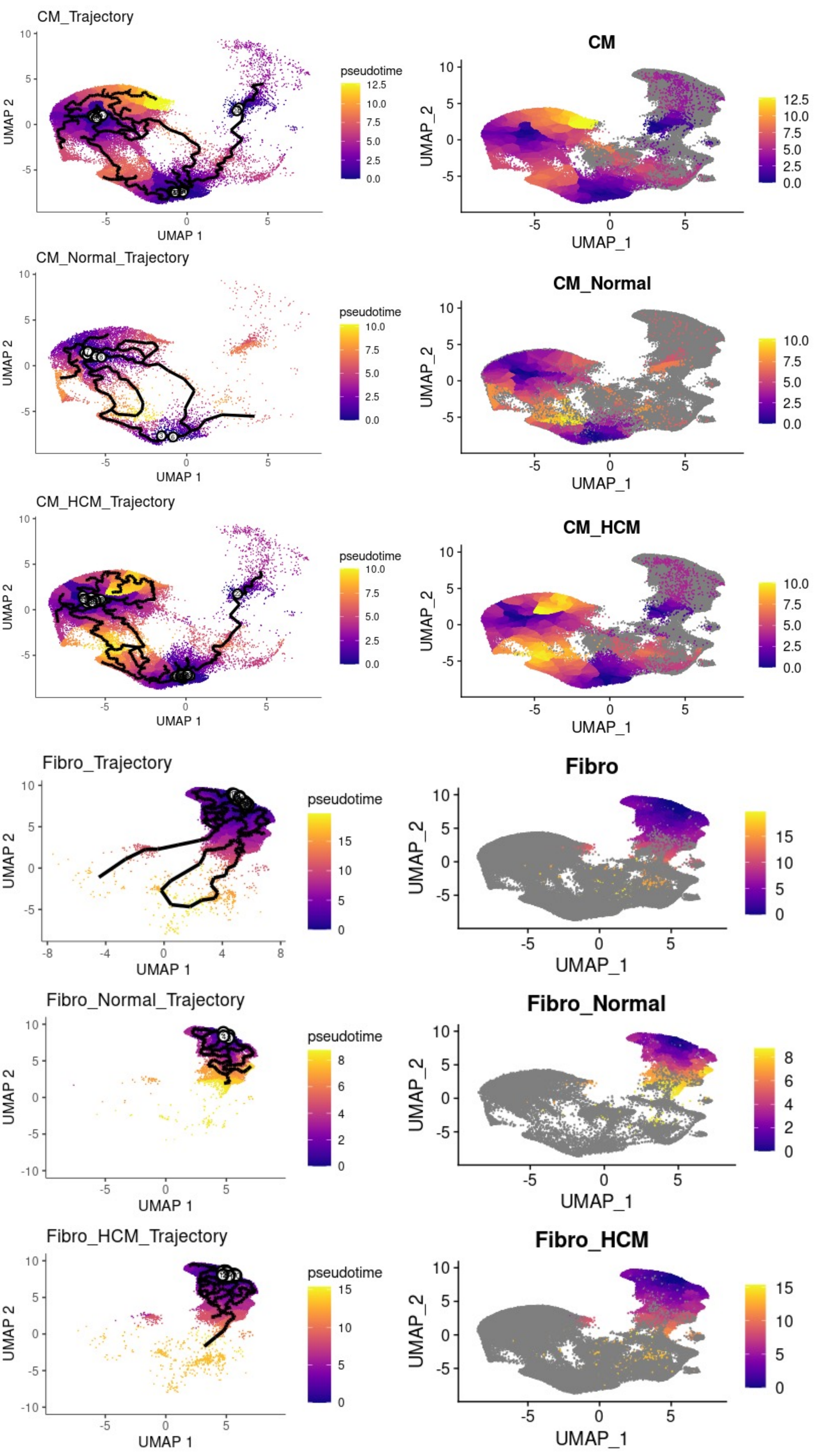

Supplemental Figure S1. Trajectory analysis of cardiomyocytes and fibroblasts in normal and HCM heart tissue. Trajectories are similar in normal and HCM cardiomyocytes and in normal and HCM fibroblasts.

Supplemental Table ST2. Number of Differentially Expressed Genes for Each Cell Type, Along Trajectory, Determined by Spatial Autocorrelation

Cell Type	Cell Class	Number of Cells	Number of Differentially Expressed Genes	Number of Gene Overlap
CM	Normal	10131	2818	1823
	HCM	57847	3589	
Fibro	Normal	6935	573	300
	HCM	22830	481	
EC	Normal	1526	661	295
	HCM	9571	614	
PC	Normal	1010	320	127
	HCM	6310	482	
Macro	Normal	2734	278	129
	HCM	5821	402	
Lymphocyte	Normal	300	325	130
	HCM	922	358	
Stromal	Normal	1350	94	28
	HCM	3895	91	
SMC	Normal	511	547	173
	HCM	1498	492	
Neuro	Normal	188	114	6
	HCM	632	137	
Lymphatic	Normal	173	105	4
	HCM	497	148	

Supplemental Table ST3. Differentially Expressed Genes Along Trajectories, Determined by Spatial Autocorrelation, Listed Alphabetically with Cell Type

	Filtered Differentially Expressed Genes Over Space	Cell Type
1	ABCA10	Endothelial
2	ABCA6	Cardiomyocyte, Pericyte
3	ABCA8	Cardiomyocyte
4	ABLIM1	Lymphatic
5	ACTA1	Neuronal
6	ACTC1	Cardiomyocyte, Lymphatic
7	ACTG1	Smooth Muscle
8	ADH1B	Cardiomyocyte, Nueronal
9	ADJ1B	Pericyte
10	AGT	Endothelial, AGT
11	AKAP13	Lymphatic
12	ALDOA	Smooth Muscle
13	ANKRD1	Lymphatic
14	ANKRD2	Smooth Muscle, Lymphatic
15	APOE	Fibroblast
16	AQP7	Macrophage
17	ASPM	Endothelial
18	ATP1A2	Lymphatic
19	B2M	Macrophage, Lymphocyte
20	C1R	Fibroblast
21	C1S	Fibroblast
22	C7	Fibroblast
23	CCL2	Neuronal
24	CCL21	Lymphatic
25	CD2	Lymphocyte
26	CD36	Smooth Muscle, Lymphatic
27	CD63	Smooth Muscle
28	CDH19	Neuronal
29	CFD	Endothelial
30	CFL1	Endothelial
31	CILP	Fibroblast
32	CMC2	Neuronal
33	CMYA5	Cardiomyocyte
34	COA7	Lymphatic
35	COL1A1	Fibroblast
36	COL6A1	Fibroblast
37	COL6A2	Fibroblast
38	COX6A2	Lymphatic
39	CPE	Endothelial
40	CSDC2	Neuronal
41	CTBS	Lymphatic
42	CYR61	Fibroblast
43	DCN	Cardiomyocyte, Endothelial
44	DCP2	Cardiomyocyte
45	DES	Cardiomyocyte
46	DNAJC8	Neuronal
47	DNM3OS	Pericyte
48	DNUFA5	Lymphatic
49	EEF1A1	Endothelial, Lymphatic
50	EHMT1	Lymphatic
51	EXOG	Smooth Muscle
52	FABP4	Smooth Muscle, Lymphatic
53	FAM134B	Lymphatic
54	FHL2	Cardiomyocyte, Lympahtic
55	FLOT1	Neuronal
56	FOS	Cardiomyocyte, Macrophage
57	FTH1	Endothelial
58	GAPDH	Smooth Muscle
59	GAS7	Lymphatic
60	GJA5	Endothelial
61	GPNMB	Neuronal
62	GPR155	Cardiomyocyte
63	GPRIN3	Cardiomyocyte
64	GRHPR	Neuronal
65	GZMK	Lymphocyte
66	H1FX	Neuronal
67	H3F3B	Smooth Muscle
68	HES1	Fibroblast
69	HIC1	Smooth Muscle
70	HLA-B	Smooth Muscle
71	HLA-DPB1	Macrophage
72	HLA-DRA	Macrophage, Lymphocyte
73	HMGN1	Lymphatic
74	HSF4	Smooth Muscle
75	ID4	Smooth Muscle
76	IDGFBP7	Lymphatic
77	IGF2	Smooth Muscle
78	IGFBP3	Endothelial
79	IGFBP7	Fibroblast
80	INHBA	Endothelial
81	IRS1	Neuronal
82	JUNB	Cardiomyocyte
83	KCNJ8	Smooth Muscle
84	KIF1C	Cardiomyocyte
85	LEPR	Endothelial
86	LGALS1	Smooth Muscle
87	LINC01089	Neuronal
88	MALAT1	Cardiomyocyte, Fibroblast, Endothelial, Macrophage, Lymphocyte, Smooth Muscle, Neuronal
89	MEG3	Cardiomyocyte, Macrophage
90	MFAP4	Fibroblast
91	MFSD14A	Neuronal
92	MPC1	Lymphatic
93	MPHOSPH8	Neuronal
94	MRRF	Lymphatic
95	MS4A6A	Cardiomyocyte
96	MYH7	Cardiomyocyte
97	MYH7B	Macrophage
98	MYL2	Cardiomyocyte, Smooth Muscle, Neuronal
99	MZT2A	Lymphatic
100	NDUFA1	Neuronal
101	NDUFB2	Neuronal
102	NEAT1	Cardiomyocyte, Pericyte
103	NEBL	Macrophage
104	NMRK2	Lymphatic
105	NOVA1	Neuronal
106	NPPA	Cardiomyocyte
107	NPPB	Cardiomyocyte
108	PDGFRB	Macrophage
109	PDLIM5	Lymphatic
110	PGM3	Neuronal
111	PKN2	Neuronal
112	PLA2G2A	Cardiomyocyte, Endothelial
113	PMEPA1	Neuronal
114	POLR2J3	Neuronal
115	POSTN	Fibroblast, Endothelial
116	PPHLN1	Neuronal
117	PPRG4	Fibroblast
118	PRKDC	Neuronal
119	PTMA	Smooth Muscle
120	PTPRS	Smooth Muscle
121	RAB8B	Smooth Muscle
122	RBFOX2	Neuronal
123	RCN2	Cardiomyocyte
124	RMND5B	Smooth Muscle
125	RP11-39404.5	Smooth Muscle
126	RP11-983P16.4	Neuronal
127	RPL10	Smooth Muscle
128	RPL11	Lymphatic
129	RPL13A	Endothelial
130	RPL23A	Endothelial
131	RPL3	Endothelial
132	RPL7	Endothelial
133	RPS15	Lymphatic
134	RRP15	Lymphatic
135	S100A1	Smooth Muscle
136	SAT1	Cardiomyocyte
137	SDHA	Lymphatic
138	SEPHS1	Neuronal
139	SERINC3	Lymphatic
140	SERPINF1	Cardiomyocyte, Fibroblast
141	SF1	Neuronal
142	SLC11A2	Neuronal
143	SLC25A6	Smooth Muscle
144	SLC35F6	Neuronal
145	SLC41A1	Lymphatic
146	SLCO5A1	Neuronal
147	SLMAP	Pericyte
148	SMIM8	Smooth Muscle
149	SMU1	Lymphatic
150	SNHG21	Neuronal
151	SORBS2	Smooth Muscle, Lymphatic
152	SREK1	Lymphatic
153	STAT6	Cardiomyocyte
154	STEAP4	Smooth Muscle
155	SYNPO	Cardiomyocyte
156	TAGLN	Smooth Muscle
157	TECRL	Smooth Muscle
158	TFPI	Smooth Muscle
159	TGFB2	Lymphatic
160	TMED4	Cardiomyocyte
161	TMEM175	Neuronal
162	TMSB4X	Endothelial, Smooth Muscle
163	TNNI3	Cardiomyocyte, Smooth Muscle
164	TNNT2	Cardiomyocyte, Lympahtic
165	TPCN1	Neuronal
166	TRMT112	Lymphatic
167	TUBA1B	Smooth Muscle
168	TYROBP	Neuronal
169	UBR3	Neuronal
170	UGDH-AS1	Neuronal
171	UTRN	Lymphatic
172	WASF2	Neuronal
173	YAP1	Neuronal
174	ZMYM2	Cardiomyocyte
175	ZNF106	Cardiomyocyte
176	ZNF211	Lymphatic

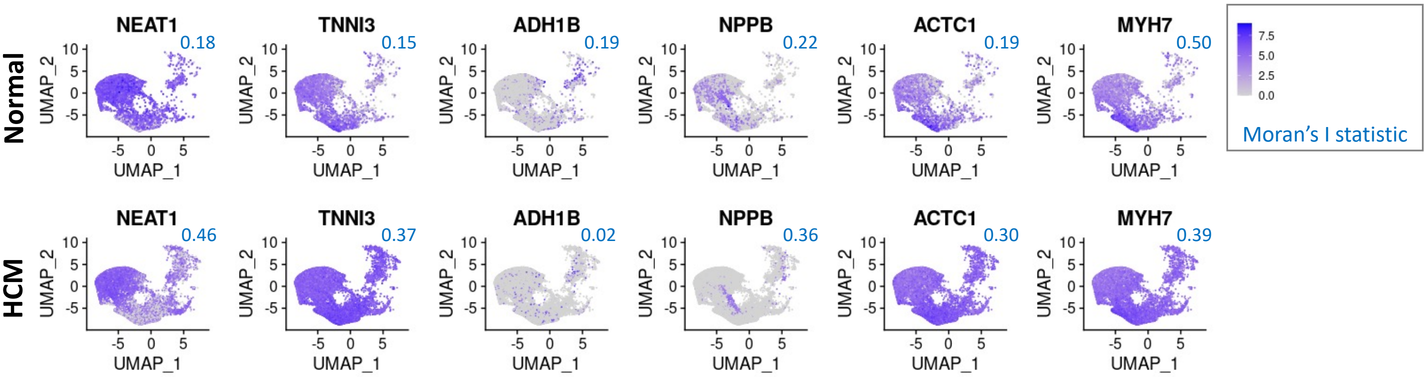

Supplemental Figure S2. Selected cardiomyocyte genes that are differentially expressed along the cardiomyocyte trajectory by spatial autocorrelation, represented in UMAP space for Normal and HCM cells

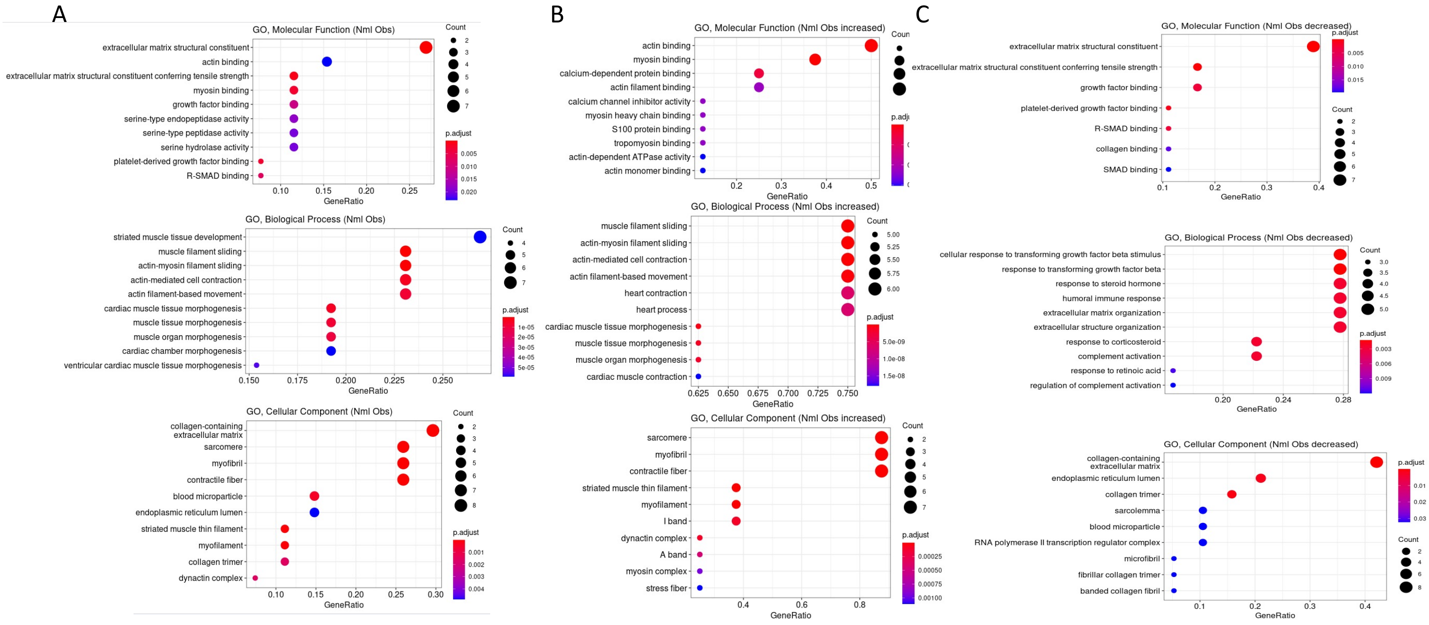

Supplemental Figure S3. Gene Ontology Enrichment Analysis of Differentially Expressed Genes in HCM. A. GO enrichment analysis of all differentially expressed genes in HCM from Table 1. B. GO enrichment analysis of Table 1 genes increased in HCM. C. GO enrichment analysis of Table 1 genes decreased in HCM.

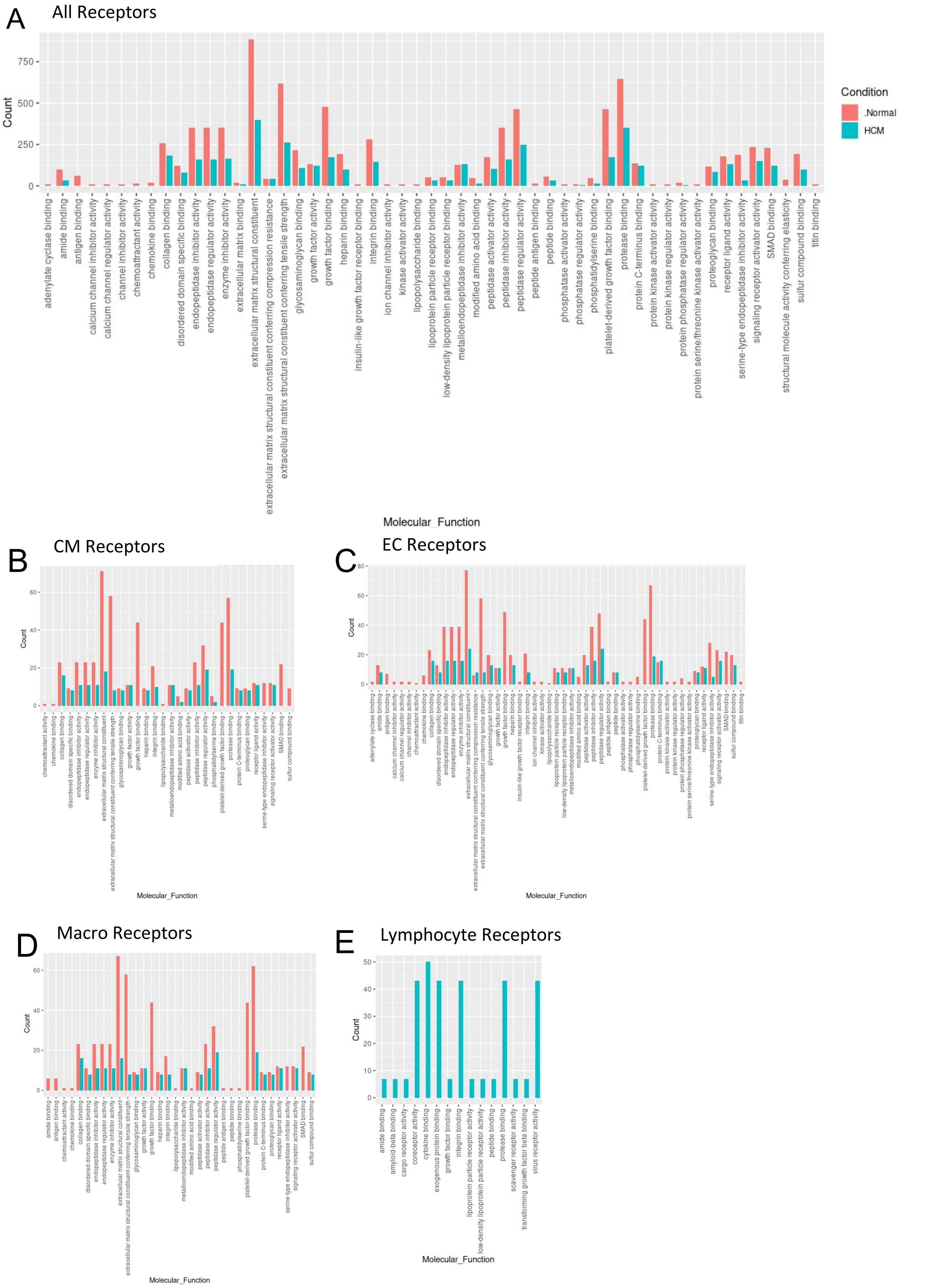

Supplemental Figure S4. Bar plot representing the total count of receptors (in expressed ligand-receptor pairs) associated with different cellular processes in Normal and HCM IVS Cells. Bar color distinguishes ligand count in normal or HCM conditions. A. Comparison of molecular processes across all cell types. B. Comparison in cardiomyocytes. C. Endothelial cells. D. Macrophages. E. Lymphocytes

Supplemental Table ST4: Fibroblast to Lymphocyte Ligand-Receptor Interactions in Normal and HCM IVS Tissue

**Normal Condition**						**HCM Condition**				
**L-Cell**	**R-Cell**	**L**	**R**	**L-R pair**		**L-Cell**	**R-Cell**	**L**	**R**	**L-R pair**
Fibro	Lymphocyte	TIMP1	CD63	TIMP1_CD63		Fibro	Lymphocyte	COL1A1	CD36	COL1A1_CD36
						Fibro	Lymphocyte	COL1A2	CD36	COL1A2_CD36
						Fibro	Lymphocyte	COL1A1	ITGB1	COL1A1_ITGB1
						Fibro	Lymphocyte	COL1A2	ITGB1	COL1A2_ITGB1
						Fibro	Lymphocyte	COL3A1	ITGB1	COL3A1_ITGB1
						Fibro	Lymphocyte	COL6A1	ITGB1	COL6A1_ITGB1
						Fibro	Lymphocyte	COL6A2	ITGB1	COL6A2_ITGB1
						Fibro	Lymphocyte	COL6A3	ITGB1	COL6A3_ITGB1
						Fibro	Lymphocyte	FBLN1	ITGB1	FBLN1_ITGB1
						Fibro	Lymphocyte	FN1	ITGB1	FN1_ITGB1
						Fibro	Lymphocyte	HSPG2	ITGB1	HSPG2_ITGB1
						Fibro	Lymphocyte	VCAN	ITGB1	VCAN_ITGB1
						Fibro	Lymphocyte	TIMP1	CD63	TIMP1_CD63

Supplemental Table ST5, Cardiomyocyte Communication Pathways No Longer Present in HCM

L-Cell	R-Cell	L	R	L-R pair
CM 2	CM 1	TIMP1	CD63	TIMP1_CD63
CM 2	CM 2	TIMP1	CD63	TIMP1_CD63
CM 2	CM 2	CALM2	INSR	CALM2_INSR
CM 2	CM 6	TIMP1	CD63	TIMP1_CD63
CM 2	CM 9	TIMP1	CD63	TIMP1_CD63
CM 2	CM 9	CALM2	INSR	CALM2_INSR
CM 2	CM 10	TIMP1	CD63	TIMP1_CD63
CM 2	Fibro 1	TIMP1	CD63	TIMP1_CD63
CM 2	Fibro 1	MFGE8	PDGFRB	MFGE8_PDGFRB
CM 2	Fibro 3	TIMP1	CD63	TIMP1_CD63
CM 2	Fibro 3	MFGE8	PDGFRB	MFGE8_PDGFRB
CM 2	Fibro 4	TIMP1	CD63	TIMP1_CD63
CM 2	Fibro 4	MFGE8	PDGFRB	MFGE8_PDGFRB
CM 2	Fibro 4	MFGE8	ITGAV	MFGE8_ITGAV
CM 2	Fibro 6	TIMP1	CD63	TIMP1_CD63
CM 2	Fibro 6	MFGE8	PDGFRB	MFGE8_PDGFRB
CM 2	Fibro 7	TIMP1	CD63	TIMP1_CD63
CM 2	Fibro 7	MFGE8	PDGFRB	MFGE8_PDGFRB
CM 2	CM 13	TIMP1	CD63	TIMP1_CD63
CM 2	CM 13	CALM2	INSR	CALM2_INSR
CM 3	Fibro 1	MFGE8	PDGFRB	MFGE8_PDGFRB
CM 3	Fibro 1	TIMP1	CD63	TIMP1_CD63
CM 3	Fibro 1	LPL	LRP1	LPL_LRP1
CM 3	Fibro 1	PSAP	LRP1	PSAP_LRP1
CM 3	Fibro 1	SERPING1	LRP1	SERPING1_LRP1
CM 3	Fibro 7	MFGE8	PDGFRB	MFGE8_PDGFRB
CM 3	Fibro 7	TIMP1	CD63	TIMP1_CD63
CM 3	Fibro 7	LPL	LRP1	LPL_LRP1
CM 3	Fibro 7	PSAP	LRP1	PSAP_LRP1
CM 3	Fibro 7	SERPING1	LRP1	SERPING1_LRP1
CM 3	Fibro 7	COL1A1	ITGB1	COL1A1_ITGB1
CM 3	Fibro 7	COL1A2	ITGB1	COL1A2_ITGB1
CM 3	Fibro 7	COL3A1	ITGB1	COL3A1_ITGB1
CM 3	Fibro 7	COL6A1	ITGB1	COL6A1_ITGB1
CM 3	Fibro 7	COL6A2	ITGB1	COL6A2_ITGB1
CM 3	Fibro 7	FN1	ITGB1	FN1_ITGB1
CM 3	Fibro 7	LAMA2	ITGB1	LAMA2_ITGB1
CM 4	CM 1	TIMP1	CD63	TIMP1_CD63
CM 4	CM 1	COL1A2	CD36	COL1A2_CD36
CM 4	CM 2	TIMP1	CD63	TIMP1_CD63
CM 4	CM 2	COL1A2	CD36	COL1A2_CD36
CM 4	CM 2	CALM2	INSR	CALM2_INSR
CM 4	CM 6	COL1A2	ITGB1	COL1A2_ITGB1
CM 4	CM 6	COL3A1	ITGB1	COL3A1_ITGB1
CM 4	CM 6	FN1	ITGB1	FN1_ITGB1
CM 4	CM 6	LAMA2	ITGB1	LAMA2_ITGB1
CM 4	CM 6	TIMP1	CD63	TIMP1_CD63
CM 4	CM 6	COL1A2	CD36	COL1A2_CD36
CM 4	CM 6	LAMA2	RPSA	LAMA2_RPSA
CM 4	CM 9	TIMP1	CD63	TIMP1_CD63
CM 4	CM 9	COL1A2	CD36	COL1A2_CD36
CM 4	CM 9	CALM2	INSR	CALM2_INSR
CM 4	CM 9	COL1A2	ITGB1	COL1A2_ITGB1
CM 4	CM 9	COL3A1	ITGB1	COL3A1_ITGB1
CM 4	CM 9	FN1	ITGB1	FN1_ITGB1
CM 4	CM 9	LAMA2	ITGB1	LAMA2_ITGB1
CM 4	CM 9	CALM2	CACNA1C	CALM2_CACNA1C
CM 4	CM 10	COL1A2	ITGB1	COL1A2_ITGB1
CM 4	CM 10	COL3A1	ITGB1	COL3A1_ITGB1
CM 4	CM 10	FN1	ITGB1	FN1_ITGB1
CM 4	CM 10	LAMA2	ITGB1	LAMA2_ITGB1
CM 4	CM 10	TIMP1	CD63	TIMP1_CD63
CM 4	CM 10	COL1A2	CD36	COL1A2_CD36
CM 4	CM 10	LAMA2	RPSA	LAMA2_RPSA
CM 4	Fibro 1	MFGE8	PDGFRB	MFGE8_PDGFRB
CM 4	Fibro 1	TIMP1	CD63	TIMP1_CD63
CM 4	Fibro 3	MFGE8	PDGFRB	MFGE8_PDGFRB
CM 4	Fibro 3	TIMP1	CD63	TIMP1_CD63
CM 4	Fibro 3	COL1A2	ITGB1	COL1A2_ITGB1
CM 4	Fibro 3	COL3A1	ITGB1	COL3A1_ITGB1
CM 4	Fibro 3	FN1	ITGB1	FN1_ITGB1
CM 4	Fibro 3	LAMA2	ITGB1	LAMA2_ITGB1
CM 4	Fibro 4	TIMP1	CD63	TIMP1_CD63
CM 4	Fibro 4	COL1A2	ITGB1	COL1A2_ITGB1
CM 4	Fibro 4	COL3A1	ITGB1	COL3A1_ITGB1
CM 4	Fibro 4	FN1	ITGB1	FN1_ITGB1
CM 4	Fibro 4	LAMA2	ITGB1	LAMA2_ITGB1
CM 4	Fibro 4	FN1	SDC2	FN1_SDC2
CM 4	Fibro 4	COL1A2	ITGAV	COL1A2_ITGAV
CM 4	Fibro 4	FN1	ITGAV	FN1_ITGAV
CM 4	Fibro 4	MFGE8	ITGAV	MFGE8_ITGAV
CM 4	Fibro 6	MFGE8	PDGFRB	MFGE8_PDGFRB
CM 4	Fibro 6	TIMP1	CD63	TIMP1_CD63
CM 4	Fibro 6	COL1A2	ITGB1	COL1A2_ITGB1
CM 4	Fibro 6	COL3A1	ITGB1	COL3A1_ITGB1
CM 4	Fibro 6	FN1	ITGB1	FN1_ITGB1
CM 4	Fibro 6	LAMA2	ITGB1	LAMA2_ITGB1
CM 4	Fibro 7	MFGE8	PDGFRB	MFGE8_PDGFRB
CM 4	Fibro 7	TIMP1	CD63	TIMP1_CD63
CM 4	Fibro 7	COL1A2	ITGB1	COL1A2_ITGB1
CM 4	Fibro 7	COL3A1	ITGB1	COL3A1_ITGB1
CM 4	Fibro 7	FN1	ITGB1	FN1_ITGB1
CM 4	Fibro 7	LAMA2	ITGB1	LAMA2_ITGB1
CM 4	CM 13	TIMP1	CD63	TIMP1_CD63
CM 4	CM 13	COL1A2	CD36	COL1A2_CD36
CM 4	CM 13	CALM2	INSR	CALM2_INSR
CM 5	Fibro 1	MFGE8	PDGFRB	MFGE8_PDGFRB
CM 5	Fibro 1	TIMP1	CD63	TIMP1_CD63
CM 5	Fibro 1	LPL	LRP1	LPL_LRP1
CM 5	Fibro 1	PSAP	LRP1	PSAP_LRP1
CM 5	Fibro 1	SERPING1	LRP1	SERPING1_LRP1
CM 5	Fibro 7	MFGE8	PDGFRB	MFGE8_PDGFRB
CM 5	Fibro 7	TIMP1	CD63	TIMP1_CD63
CM 5	Fibro 7	LPL	LRP1	LPL_LRP1
CM 5	Fibro 7	PSAP	LRP1	PSAP_LRP1
CM 5	Fibro 7	SERPING1	LRP1	SERPING1_LRP1
CM 5	Fibro 7	COL1A1	ITGB1	COL1A1_ITGB1
CM 5	Fibro 7	COL1A2	ITGB1	COL1A2_ITGB1
CM 5	Fibro 7	COL3A1	ITGB1	COL3A1_ITGB1
CM 5	Fibro 7	COL6A1	ITGB1	COL6A1_ITGB1
CM 5	Fibro 7	COL6A2	ITGB1	COL6A2_ITGB1
CM 5	Fibro 7	FN1	ITGB1	FN1_ITGB1
CM 5	Fibro 7	LAMA2	ITGB1	LAMA2_ITGB1
CM 5	Fibro 7	VEGFA	ITGB1	VEGFA_ITGB1
CM 7	CM 1	COL1A1	CD36	COL1A1_CD36
CM 7	CM 1	COL1A2	CD36	COL1A2_CD36
CM 7	CM 1	TIMP1	CD63	TIMP1_CD63
CM 7	CM 2	COL1A1	CD36	COL1A1_CD36
CM 7	CM 2	COL1A2	CD36	COL1A2_CD36
CM 7	CM 2	TIMP1	CD63	TIMP1_CD63
CM 7	CM 2	CALM2	INSR	CALM2_INSR
CM 7	CM 6	COL1A1	CD36	COL1A1_CD36
CM 7	CM 6	COL1A2	CD36	COL1A2_CD36
CM 7	CM 6	TIMP1	CD63	TIMP1_CD63
CM 7	CM 6	COL1A1	ITGB1	COL1A1_ITGB1
CM 7	CM 6	COL1A2	ITGB1	COL1A2_ITGB1
CM 7	CM 6	COL3A1	ITGB1	COL3A1_ITGB1
CM 7	CM 6	COL6A1	ITGB1	COL6A1_ITGB1
CM 7	CM 6	COL6A2	ITGB1	COL6A2_ITGB1
CM 7	CM 6	FN1	ITGB1	FN1_ITGB1
CM 7	CM 6	LAMA2	ITGB1	LAMA2_ITGB1
CM 7	CM 6	LAMA2	RPSA	LAMA2_RPSA
CM 7	CM 9	CALM2	CACNA1C	CALM2_CACNA1C
CM 7	CM 9	COL1A1	CD36	COL1A1_CD36
CM 7	CM 9	COL1A2	CD36	COL1A2_CD36
CM 7	CM 9	TIMP1	CD63	TIMP1_CD63
CM 7	CM 9	CALM2	INSR	CALM2_INSR
CM 7	CM 9	COL1A1	ITGB1	COL1A1_ITGB1
CM 7	CM 9	COL1A2	ITGB1	COL1A2_ITGB1
CM 7	CM 9	COL3A1	ITGB1	COL3A1_ITGB1
CM 7	CM 9	COL6A1	ITGB1	COL6A1_ITGB1
CM 7	CM 9	COL6A2	ITGB1	COL6A2_ITGB1
CM 7	CM 9	FN1	ITGB1	FN1_ITGB1
CM 7	CM 9	LAMA2	ITGB1	LAMA2_ITGB1
CM 7	CM 10	COL1A1	CD36	COL1A1_CD36
CM 7	CM 10	COL1A2	CD36	COL1A2_CD36
CM 7	CM 10	TIMP1	CD63	TIMP1_CD63
CM 7	CM 10	COL1A1	ITGB1	COL1A1_ITGB1
CM 7	CM 10	COL1A2	ITGB1	COL1A2_ITGB1
CM 7	CM 10	COL3A1	ITGB1	COL3A1_ITGB1
CM 7	CM 10	COL6A1	ITGB1	COL6A1_ITGB1
CM 7	CM 10	COL6A2	ITGB1	COL6A2_ITGB1
CM 7	CM 10	FN1	ITGB1	FN1_ITGB1
CM 7	CM 10	LAMA2	ITGB1	LAMA2_ITGB1
CM 7	CM 10	LAMA2	RPSA	LAMA2_RPSA
CM 7	Fibro 1	TIMP1	CD63	TIMP1_CD63
CM 7	Fibro 1	MFGE8	PDGFRB	MFGE8_PDGFRB
CM 7	Fibro 1	PSAP	LRP1	PSAP_LRP1
CM 7	Fibro 3	TIMP1	CD63	TIMP1_CD63
CM 7	Fibro 3	MFGE8	PDGFRB	MFGE8_PDGFRB
CM 7	Fibro 3	PSAP	LRP1	PSAP_LRP1
CM 7	Fibro 3	COL1A1	ITGB1	COL1A1_ITGB1
CM 7	Fibro 3	COL1A2	ITGB1	COL1A2_ITGB1
CM 7	Fibro 3	COL3A1	ITGB1	COL3A1_ITGB1
CM 7	Fibro 3	COL6A1	ITGB1	COL6A1_ITGB1
CM 7	Fibro 3	COL6A2	ITGB1	COL6A2_ITGB1
CM 7	Fibro 3	FN1	ITGB1	FN1_ITGB1
CM 7	Fibro 3	LAMA2	ITGB1	LAMA2_ITGB1
CM 7	Fibro 4	TIMP1	CD63	TIMP1_CD63
CM 7	Fibro 4	MFGE8	PDGFRB	MFGE8_PDGFRB
CM 7	Fibro 4	PSAP	LRP1	PSAP_LRP1
CM 7	Fibro 4	COL1A1	ITGB1	COL1A1_ITGB1
CM 7	Fibro 4	COL1A2	ITGB1	COL1A2_ITGB1
CM 7	Fibro 4	COL3A1	ITGB1	COL3A1_ITGB1
CM 7	Fibro 4	COL6A1	ITGB1	COL6A1_ITGB1
CM 7	Fibro 4	COL6A2	ITGB1	COL6A2_ITGB1
CM 7	Fibro 4	FN1	ITGB1	FN1_ITGB1
CM 7	Fibro 4	LAMA2	ITGB1	LAMA2_ITGB1
CM 7	Fibro 4	FN1	SDC2	FN1_SDC2
CM 7	Fibro 4	COL1A1	ITGAV	COL1A1_ITGAV
CM 7	Fibro 4	COL1A2	ITGAV	COL1A2_ITGAV
CM 7	Fibro 4	FN1	ITGAV	FN1_ITGAV
CM 7	Fibro 4	MFGE8	ITGAV	MFGE8_ITGAV
CM 7	Fibro 6	TIMP1	CD63	TIMP1_CD63
CM 7	Fibro 6	MFGE8	PDGFRB	MFGE8_PDGFRB
CM 7	Fibro 6	PSAP	LRP1	PSAP_LRP1
CM 7	Fibro 6	COL1A1	ITGB1	COL1A1_ITGB1
CM 7	Fibro 6	COL1A2	ITGB1	COL1A2_ITGB1
CM 7	Fibro 6	COL3A1	ITGB1	COL3A1_ITGB1
CM 7	Fibro 6	COL6A1	ITGB1	COL6A1_ITGB1
CM 7	Fibro 6	COL6A2	ITGB1	COL6A2_ITGB1
CM 7	Fibro 6	FN1	ITGB1	FN1_ITGB1
CM 7	Fibro 6	LAMA2	ITGB1	LAMA2_ITGB1
CM 7	Fibro 7	TIMP1	CD63	TIMP1_CD63
CM 7	Fibro 7	MFGE8	PDGFRB	MFGE8_PDGFRB
CM 7	Fibro 7	PSAP	LRP1	PSAP_LRP1
CM 7	Fibro 7	COL1A1	ITGB1	COL1A1_ITGB1
CM 7	Fibro 7	COL1A2	ITGB1	COL1A2_ITGB1
CM 7	Fibro 7	COL3A1	ITGB1	COL3A1_ITGB1
CM 7	Fibro 7	COL6A1	ITGB1	COL6A1_ITGB1
CM 7	Fibro 7	COL6A2	ITGB1	COL6A2_ITGB1
CM 7	Fibro 7	FN1	ITGB1	FN1_ITGB1
CM 7	Fibro 7	LAMA2	ITGB1	LAMA2_ITGB1
CM 7	CM 13	TIMP1	CD63	TIMP1_CD63
CM 7	CM 13	COL1A1	CD36	COL1A1_CD36
CM 7	CM 13	COL1A2	CD36	COL1A2_CD36
CM 7	CM 13	CALM2	INSR	CALM2_INSR
CM 11	CM 1	COL1A1	CD36	COL1A1_CD36
CM 11	CM 1	COL1A2	CD36	COL1A2_CD36
CM 11	CM 1	TIMP1	CD63	TIMP1_CD63
CM 11	CM 2	COL1A1	CD36	COL1A1_CD36
CM 11	CM 2	COL1A2	CD36	COL1A2_CD36
CM 11	CM 2	TIMP1	CD63	TIMP1_CD63
CM 11	CM 2	CALM2	INSR	CALM2_INSR
CM 11	CM 2	SORBS1	INSR	SORBS1_INSR
CM 11	CM 6	COL1A1	CD36	COL1A1_CD36
CM 11	CM 6	COL1A2	CD36	COL1A2_CD36
CM 11	CM 6	TIMP1	CD63	TIMP1_CD63
CM 11	CM 6	ADAM17	ITGB1	ADAM17_ITGB1
CM 11	CM 6	COL1A1	ITGB1	COL1A1_ITGB1
CM 11	CM 6	COL1A2	ITGB1	COL1A2_ITGB1
CM 11	CM 6	COL3A1	ITGB1	COL3A1_ITGB1
CM 11	CM 6	COL6A1	ITGB1	COL6A1_ITGB1
CM 11	CM 6	COL6A2	ITGB1	COL6A2_ITGB1
CM 11	CM 6	FN1	ITGB1	FN1_ITGB1
CM 11	CM 6	LAMA2	RPSA	LAMA2_RPSA
CM 11	CM 9	COL1A1	CD36	COL1A1_CD36
CM 11	CM 9	COL1A2	CD36	COL1A2_CD36
CM 11	CM 9	TIMP1	CD63	TIMP1_CD63
CM 11	CM 9	CALM2	INSR	CALM2_INSR
CM 11	CM 9	SORBS1	INSR	SORBS1_INSR
CM 11	CM 9	ADAM17	ITGB1	ADAM17_ITGB1
CM 11	CM 9	COL1A1	ITGB1	COL1A1_ITGB1
CM 11	CM 9	COL1A2	ITGB1	COL1A2_ITGB1
CM 11	CM 9	COL3A1	ITGB1	COL3A1_ITGB1
CM 11	CM 9	COL6A1	ITGB1	COL6A1_ITGB1
CM 11	CM 9	COL6A2	ITGB1	COL6A2_ITGB1
CM 11	CM 9	FN1	ITGB1	FN1_ITGB1
CM 11	CM 9	LAMA2	ITGB1	LAMA2_ITGB1
CM 11	CM 9	CALM2	CACNA1C	CALM2_CACNA1C
CM 11	CM 10	COL1A1	CD36	COL1A1_CD36
CM 11	CM 10	COL1A2	CD36	COL1A2_CD36
CM 11	CM 10	TIMP1	CD63	TIMP1_CD63
CM 11	CM 10	ADAM17	ITGB1	ADAM17_ITGB1
CM 11	CM 10	COL1A1	ITGB1	COL1A1_ITGB1
CM 11	CM 10	COL1A2	ITGB1	COL1A2_ITGB1
CM 11	CM 10	COL3A1	ITGB1	COL3A1_ITGB1
CM 11	CM 10	COL6A1	ITGB1	COL6A1_ITGB1
CM 11	CM 10	COL6A2	ITGB1	COL6A2_ITGB1
CM 11	CM 10	FN1	ITGB1	FN1_ITGB1
CM 11	CM 10	LAMA2	RPSA	LAMA2_RPSA
CM 11	Fibro 1	MFGE8	PDGFRB	MFGE8_PDGFRB
CM 11	Fibro 1	LPL	LRP1	LPL_LRP1
CM 11	Fibro 1	PSAP	LRP1	PSAP_LRP1
CM 11	Fibro 1	SERPING1	LRP1	SERPING1_LRP1
CM 11	Fibro 1	TIMP1	CD63	TIMP1_CD63
CM 11	Fibro 3	MFGE8	PDGFRB	MFGE8_PDGFRB
CM 11	Fibro 3	LPL	LRP1	LPL_LRP1
CM 11	Fibro 3	PSAP	LRP1	PSAP_LRP1
CM 11	Fibro 3	SERPING1	LRP1	SERPING1_LRP1
CM 11	Fibro 3	TIMP1	CD63	TIMP1_CD63
CM 11	Fibro 3	ADAM17	ITGB1	ADAM17_ITGB1
CM 11	Fibro 3	COL1A1	ITGB1	COL1A1_ITGB1
CM 11	Fibro 3	COL1A2	ITGB1	COL1A2_ITGB1
CM 11	Fibro 3	COL3A1	ITGB1	COL3A1_ITGB1
CM 11	Fibro 3	COL6A1	ITGB1	COL6A1_ITGB1
CM 11	Fibro 3	COL6A2	ITGB1	COL6A2_ITGB1
CM 11	Fibro 3	FN1	ITGB1	FN1_ITGB1
CM 11	Fibro 4	MFGE8	PDGFRB	MFGE8_PDGFRB
CM 11	Fibro 4	LPL	LRP1	LPL_LRP1
CM 11	Fibro 4	PSAP	LRP1	PSAP_LRP1
CM 11	Fibro 4	SERPING1	LRP1	SERPING1_LRP1
CM 11	Fibro 4	TIMP1	CD63	TIMP1_CD63
CM 11	Fibro 4	ADAM17	ITGB1	ADAM17_ITGB1
CM 11	Fibro 4	COL1A1	ITGB1	COL1A1_ITGB1
CM 11	Fibro 4	COL1A2	ITGB1	COL1A2_ITGB1
CM 11	Fibro 4	COL3A1	ITGB1	COL3A1_ITGB1
CM 11	Fibro 4	COL6A1	ITGB1	COL6A1_ITGB1
CM 11	Fibro 4	COL6A2	ITGB1	COL6A2_ITGB1
CM 11	Fibro 4	FN1	ITGB1	FN1_ITGB1
CM 11	Fibro 4	COL1A1	ITGAV	COL1A1_ITGAV
CM 11	Fibro 4	COL1A2	ITGAV	COL1A2_ITGAV
CM 11	Fibro 4	FN1	ITGAV	FN1_ITGAV
CM 11	Fibro 4	MFGE8	ITGAV	MFGE8_ITGAV
CM 11	Fibro 4	FN1	SDC2	FN1_SDC2
CM 11	Fibro 6	MFGE8	PDGFRB	MFGE8_PDGFRB
CM 11	Fibro 6	LPL	LRP1	LPL_LRP1
CM 11	Fibro 6	PSAP	LRP1	PSAP_LRP1
CM 11	Fibro 6	SERPING1	LRP1	SERPING1_LRP1
CM 11	Fibro 6	TIMP1	CD63	TIMP1_CD63
CM 11	Fibro 6	ADAM17	ITGB1	ADAM17_ITGB1
CM 11	Fibro 6	COL1A1	ITGB1	COL1A1_ITGB1
CM 11	Fibro 6	COL1A2	ITGB1	COL1A2_ITGB1
CM 11	Fibro 6	COL3A1	ITGB1	COL3A1_ITGB1
CM 11	Fibro 6	COL6A1	ITGB1	COL6A1_ITGB1
CM 11	Fibro 6	COL6A2	ITGB1	COL6A2_ITGB1
CM 11	Fibro 6	FN1	ITGB1	FN1_ITGB1
CM 11	Fibro 7	MFGE8	PDGFRB	MFGE8_PDGFRB
CM 11	Fibro 7	LPL	LRP1	LPL_LRP1
CM 11	Fibro 7	PSAP	LRP1	PSAP_LRP1
CM 11	Fibro 7	SERPING1	LRP1	SERPING1_LRP1
CM 11	Fibro 7	TIMP1	CD63	TIMP1_CD63
CM 11	Fibro 7	ADAM17	ITGB1	ADAM17_ITGB1
CM 11	Fibro 7	COL1A1	ITGB1	COL1A1_ITGB1
CM 11	Fibro 7	COL1A2	ITGB1	COL1A2_ITGB1
CM 11	Fibro 7	COL3A1	ITGB1	COL3A1_ITGB1
CM 11	Fibro 7	COL6A1	ITGB1	COL6A1_ITGB1
CM 11	Fibro 7	COL6A2	ITGB1	COL6A2_ITGB1
CM 11	Fibro 7	FN1	ITGB1	FN1_ITGB1
CM 11	CM 13	COL1A1	CD36	COL1A1_CD36
CM 11	CM 13	COL1A2	CD36	COL1A2_CD36
CM 11	CM 13	TIMP1	CD63	TIMP1_CD63
CM 11	CM 13	CALM2	INSR	CALM2_INSR
CM 11	CM 13	SORBS1	INSR	SORBS1_INSR

Supplemental Table ST6. Ligands Lost in HCM Fibroblast Cluster 4 and Associated Molecular Functions

	**Ligands lost in HCM fibro 4 condition**																								
**Molecular Function**	C3	CALR	COL18A1	COL4A1	COL5A1	COL5A2	CXCL12	CYR61	FBLN1	FBN1	HSP90B1	IGF1	LAMA2	LAMB1	LAMC1	LGALS3BP	LRPAP1	NID1	PDGFD	PTN	TFPI	THBS2	TIMP2	TNC	**Count**
amide binding		x															x								2
collagen binding																		x							1
endopeptidase inhibitor activity	x																				x		x		3
endopeptidase regulator activity	x																				x		x		3
enzyme inhibitor activity	x																			x	x		x		4
extracellular matrix binding																		x							1
extracellular matrix structural constituent			x	x	x	x			x					x				x							7
extracellular matrix structural constituent conferring tensile strength			x	x	x	x							x												5
glycosaminoglycan binding					x												x			x					3
growth factor activity												x								x					2
growth factor binding				x	x	x														x					4
heparin binding					x												x			x					3
insulin-like growth factor receptor binding												x													1
integrin binding		x			x							x		x						x			x		6
lipoprotein particle receptor binding											x						x								2
low-density lipoprotein particle receptor binding											x						x								2
metalloendopeptidase inhibitor activity																							x		1
peptidase activator activity									x																1
peptidase inhibitor activity	x																				x		x		3
peptidase regulator activity	x								x												x		x		4
peptide binding		x															x								2
phosphatase regulator activity																				x					1
platelet-derived growth factor binding				x	x																				2
protease binding																							x		1
protein C-terminus binding									x																1
protein phosphatase regulator activity																				x					1
proteoglycan binding					x													x		x					3
receptor ligand activity												x					x			x					3
signaling receptor activator activity												x					x			x					3
SMAD binding						x																			1
sulfur compound binding					x												x			x					3

Supplemental Table ST7. Reduction in Fibroblast Cluster 4 Ligand-Receptor Interactions in HCM

Ligand	Receptor	L-R pairs	HCM
Fibro 4	Fibro 3	41	10
Fibro 2	Fibro 4	51	18
Fibro 4	Fibro 8	39	4
Fibro 4	Fibro 7	39	3
Fibro 4	Fibro 4	54	12

Supplemental Table ST8. Ligand-Receptor Interactions that are Increased in HCM between Fibroblast Subtypes and Cardiomyocyte Subtypes

Normal

L-Cell	R-Cell	L	R	L-R pair
Fibro 2	CM 1	C3	CD81	C3_CD81
Fibro 2	CM 1	TIMP1	CD63	TIMP1_CD63
Fibro 2	CM 1	COL1A1	CD36	COL1A1_CD36
Fibro 2	CM 1	COL1A2	CD36	COL1A2_CD36
Fibro 3	CM 1	C3	CD81	C3_CD81
Fibro 3	CM 1	TIMP1	CD63	TIMP1_CD63
Fibro 3	CM 1	COL1A1	CD36	COL1A1_CD36
Fibro 3	CM 1	COL1A2	CD36	COL1A2_CD36
Fibro 4	CM 1	C3	CD81	C3_CD81
Fibro 4	CM 1	TIMP1	CD63	TIMP1_CD63
Fibro 4	CM 1	COL1A1	CD36	COL1A1_CD36
Fibro 4	CM 1	COL1A2	CD36	COL1A2_CD36
Fibro 5	CM 1	C3	CD81	C3_CD81
Fibro 5	CM 1	TIMP1	CD63	TIMP1_CD63
Fibro 5	CM 1	COL1A1	CD36	COL1A1_CD36
Fibro 5	CM 1	COL1A2	CD36	COL1A2_CD36
Fibro 2	CM 2	TIMP1	CD63	TIMP1_CD63
Fibro 2	CM 2	COL1A1	CD36	COL1A1_CD36
Fibro 2	CM 2	COL1A2	CD36	COL1A2_CD36
Fibro 2	CM 2	CALM2	INSR	CALM2_INSR
Fibro 2	CM 2	IGF1	INSR	IGF1_INSR
Fibro 3	CM 2	TIMP1	CD63	TIMP1_CD63
Fibro 3	CM 2	COL1A1	CD36	COL1A1_CD36
Fibro 3	CM 2	COL1A2	CD36	COL1A2_CD36
Fibro 3	CM 2	CALM2	INSR	CALM2_INSR
Fibro 3	CM 2	IGF1	INSR	IGF1_INSR
Fibro 4	CM 2	TIMP1	CD63	TIMP1_CD63
Fibro 4	CM 2	COL1A1	CD36	COL1A1_CD36
Fibro 4	CM 2	COL1A2	CD36	COL1A2_CD36
Fibro 4	CM 2	CALM2	INSR	CALM2_INSR
Fibro 4	CM 2	IGF1	INSR	IGF1_INSR
Fibro 5	CM 2	TIMP1	CD63	TIMP1_CD63
Fibro 5	CM 2	COL1A1	CD36	COL1A1_CD36
Fibro 5	CM 2	COL1A2	CD36	COL1A2_CD36
Fibro 5	CM 2	CALM2	INSR	CALM2_INSR
Fibro 5	CM 2	IGF1	INSR	IGF1_INSR

HCM

L-Cell	R-Cell	L	R	L-R pair
Fibro 2	CM 1	COL1A1	CD36	COL1A1_CD36
Fibro 2	CM 1	COL1A2	CD36	COL1A2_CD36
Fibro 2	CM 1	COL1A1	ITGB1	COL1A1_ITGB1
Fibro 2	CM 1	COL1A2	ITGB1	COL1A2_ITGB1
Fibro 2	CM 1	COL3A1	ITGB1	COL3A1_ITGB1
Fibro 2	CM 1	COL6A1	ITGB1	COL6A1_ITGB1
Fibro 2	CM 1	COL6A2	ITGB1	COL6A2_ITGB1
Fibro 2	CM 1	COL6A3	ITGB1	COL6A3_ITGB1
Fibro 2	CM 1	FBLN1	ITGB1	FBLN1_ITGB1
Fibro 2	CM 1	FBN1	ITGB1	FBN1_ITGB1
Fibro 2	CM 1	FN1	ITGB1	FN1_ITGB1
Fibro 2	CM 1	HSPG2	ITGB1	HSPG2_ITGB1
Fibro 2	CM 1	LAMA2	ITGB1	LAMA2_ITGB1
Fibro 2	CM 1	LAMC1	ITGB1	LAMC1_ITGB1
Fibro 2	CM 1	LGALS3BP	ITGB1	LGALS3BP_ITGB1
Fibro 2	CM 1	TIMP2	ITGB1	TIMP2_ITGB1
Fibro 2	CM 1	VCAN	ITGB1	VCAN_ITGB1
Fibro 2	CM 1	TIMP1	CD63	TIMP1_CD63
Fibro 3	CM 1	COL1A2	CD36	COL1A2_CD36
Fibro 3	CM 1	COL1A2	ITGB1	COL1A2_ITGB1
Fibro 3	CM 1	COL3A1	ITGB1	COL3A1_ITGB1
Fibro 3	CM 1	COL6A1	ITGB1	COL6A1_ITGB1
Fibro 3	CM 1	COL6A2	ITGB1	COL6A2_ITGB1
Fibro 3	CM 1	COL6A3	ITGB1	COL6A3_ITGB1
Fibro 3	CM 1	FBLN1	ITGB1	FBLN1_ITGB1
Fibro 3	CM 1	FN1	ITGB1	FN1_ITGB1
Fibro 3	CM 1	HSPG2	ITGB1	HSPG2_ITGB1
Fibro 3	CM 1	LAMC1	ITGB1	LAMC1_ITGB1
Fibro 3	CM 1	VCAN	ITGB1	VCAN_ITGB1
Fibro 3	CM 1	TIMP1	CD63	TIMP1_CD63
Fibro 4	CM 1	COL1A1	CD36	COL1A1_CD36
Fibro 4	CM 1	COL1A2	CD36	COL1A2_CD36
Fibro 4	CM 1	COL1A1	ITGB1	COL1A1_ITGB1
Fibro 4	CM 1	COL1A2	ITGB1	COL1A2_ITGB1
Fibro 4	CM 1	COL3A1	ITGB1	COL3A1_ITGB1
Fibro 4	CM 1	COL6A1	ITGB1	COL6A1_ITGB1
Fibro 4	CM 1	COL6A2	ITGB1	COL6A2_ITGB1
Fibro 4	CM 1	COL6A3	ITGB1	COL6A3_ITGB1
Fibro 4	CM 1	FN1	ITGB1	FN1_ITGB1
Fibro 4	CM 1	HSPG2	ITGB1	HSPG2_ITGB1
Fibro 4	CM 1	VCAN	ITGB1	VCAN_ITGB1
Fibro 4	CM 1	TIMP1	CD63	TIMP1_CD63
Fibro 5	CM 1	COL1A1	CD36	COL1A1_CD36
Fibro 5	CM 1	COL1A2	CD36	COL1A2_CD36
Fibro 5	CM 1	COL1A1	ITGB1	COL1A1_ITGB1
Fibro 5	CM 1	COL1A2	ITGB1	COL1A2_ITGB1
Fibro 5	CM 1	COL3A1	ITGB1	COL3A1_ITGB1
Fibro 5	CM 1	COL6A1	ITGB1	COL6A1_ITGB1
Fibro 5	CM 1	COL6A2	ITGB1	COL6A2_ITGB1
Fibro 5	CM 1	COL6A3	ITGB1	COL6A3_ITGB1
Fibro 5	CM 1	FN1	ITGB1	FN1_ITGB1
Fibro 5	CM 1	HSPG2	ITGB1	HSPG2_ITGB1
Fibro 5	CM 1	LAMA2	ITGB1	LAMA2_ITGB1
Fibro 5	CM 1	LAMB1	ITGB1	LAMB1_ITGB1
Fibro 5	CM 1	LAMC1	ITGB1	LAMC1_ITGB1
Fibro 5	CM 1	VCAN	ITGB1	VCAN_ITGB1
Fibro 5	CM 1	TIMP1	CD63	TIMP1_CD63
Fibro 2	CM 2	COL1A1	CD36	COL1A1_CD36
Fibro 2	CM 2	COL1A2	CD36	COL1A2_CD36
Fibro 2	CM 2	COL1A1	ITGB1	COL1A1_ITGB1
Fibro 2	CM 2	COL1A2	ITGB1	COL1A2_ITGB1
Fibro 2	CM 2	COL3A1	ITGB1	COL3A1_ITGB1
Fibro 2	CM 2	COL6A1	ITGB1	COL6A1_ITGB1
Fibro 2	CM 2	COL6A2	ITGB1	COL6A2_ITGB1
Fibro 2	CM 2	COL6A3	ITGB1	COL6A3_ITGB1
Fibro 2	CM 2	FBLN1	ITGB1	FBLN1_ITGB1
Fibro 2	CM 2	FBN1	ITGB1	FBN1_ITGB1
Fibro 2	CM 2	FN1	ITGB1	FN1_ITGB1
Fibro 2	CM 2	HSPG2	ITGB1	HSPG2_ITGB1
Fibro 2	CM 2	LAMA2	ITGB1	LAMA2_ITGB1
Fibro 2	CM 2	LAMC1	ITGB1	LAMC1_ITGB1
Fibro 2	CM 2	LGALS3BP	ITGB1	LGALS3BP_ITGB1
Fibro 2	CM 2	TIMP2	ITGB1	TIMP2_ITGB1
Fibro 2	CM 2	VCAN	ITGB1	VCAN_ITGB1
Fibro 2	CM 2	TIMP1	CD63	TIMP1_CD63
Fibro 3	CM 2	COL1A2	CD36	COL1A2_CD36
Fibro 3	CM 2	COL1A2	ITGB1	COL1A2_ITGB1
Fibro 3	CM 2	COL3A1	ITGB1	COL3A1_ITGB1
Fibro 3	CM 2	COL6A1	ITGB1	COL6A1_ITGB1
Fibro 3	CM 2	COL6A2	ITGB1	COL6A2_ITGB1
Fibro 3	CM 2	COL6A3	ITGB1	COL6A3_ITGB1
Fibro 3	CM 2	FBLN1	ITGB1	FBLN1_ITGB1
Fibro 3	CM 2	FN1	ITGB1	FN1_ITGB1
Fibro 3	CM 2	HSPG2	ITGB1	HSPG2_ITGB1
Fibro 3	CM 2	LAMC1	ITGB1	LAMC1_ITGB1
Fibro 3	CM 2	VCAN	ITGB1	VCAN_ITGB1
Fibro 3	CM 2	TIMP1	CD63	TIMP1_CD63
Fibro 4	CM 2	COL1A1	CD36	COL1A1_CD36
Fibro 4	CM 2	COL1A2	CD36	COL1A2_CD36
Fibro 4	CM 2	COL1A1	ITGB1	COL1A1_ITGB1
Fibro 4	CM 2	COL1A2	ITGB1	COL1A2_ITGB1
Fibro 4	CM 2	COL3A1	ITGB1	COL3A1_ITGB1
Fibro 4	CM 2	COL6A1	ITGB1	COL6A1_ITGB1
Fibro 4	CM 2	COL6A2	ITGB1	COL6A2_ITGB1
Fibro 4	CM 2	COL6A3	ITGB1	COL6A3_ITGB1
Fibro 4	CM 2	FN1	ITGB1	FN1_ITGB1
Fibro 4	CM 2	HSPG2	ITGB1	HSPG2_ITGB1
Fibro 4	CM 2	VCAN	ITGB1	VCAN_ITGB1
Fibro 4	CM 2	TIMP1	CD63	TIMP1_CD63
Fibro 5	CM 2	COL1A1	CD36	COL1A1_CD36
Fibro 5	CM 2	COL1A2	CD36	COL1A2_CD36
Fibro 5	CM 2	COL1A1	ITGB1	COL1A1_ITGB1
Fibro 5	CM 2	COL1A2	ITGB1	COL1A2_ITGB1
Fibro 5	CM 2	COL3A1	ITGB1	COL3A1_ITGB1
Fibro 5	CM 2	COL6A1	ITGB1	COL6A1_ITGB1
Fibro 5	CM 2	COL6A2	ITGB1	COL6A2_ITGB1
Fibro 5	CM 2	COL6A3	ITGB1	COL6A3_ITGB1
Fibro 5	CM 2	FN1	ITGB1	FN1_ITGB1
Fibro 5	CM 2	HSPG2	ITGB1	HSPG2_ITGB1
Fibro 5	CM 2	LAMA2	ITGB1	LAMA2_ITGB1
Fibro 5	CM 2	LAMB1	ITGB1	LAMB1_ITGB1
Fibro 5	CM 2	LAMC1	ITGB1	LAMC1_ITGB1
Fibro 5	CM 2	VCAN	ITGB1	VCAN_ITGB1

Supplemental Table ST9. Ligand-Receptor Interactions that are Increased in HCM between Cardiomyocyte Cluster 8 and Cardiomyocyte Subtypes and Myofibroblasts

Normal						HCM				
**L-Cell**	**R-Cell**	**L**	**R**	**L-R pair**		**L-Cell**	**R-Cell**	**L**	**R**	**L-R pair**
CM 8	CM 1	COL1A1	CD36	COL1A1_CD36		CM 8	CM 1	COL1A2	CD36	COL1A2_CD36
CM 8	CM 1	COL1A2	CD36	COL1A2_CD36		CM 8	CM 1	TIMP1	CD63	TIMP1_CD63
CM 8	CM 1	TIMP1	CD63	TIMP1_CD63		CM 8	CM 1	COL1A2	ITGB1	COL1A2_ITGB1
CM 8	CM 1	C3	CD81	C3_CD81		CM 8	CM 1	COL3A1	ITGB1	COL3A1_ITGB1
CM 8	CM 2	COL1A1	CD36	COL1A1_CD36		CM 8	CM 1	COL6A1	ITGB1	COL6A1_ITGB1
CM 8	CM 2	COL1A2	CD36	COL1A2_CD36		CM 8	CM 1	COL6A2	ITGB1	COL6A2_ITGB1
CM 8	CM 2	TIMP1	CD63	TIMP1_CD63		CM 8	CM 1	FN1	ITGB1	FN1_ITGB1
CM 8	CM 2	CALM2	INSR	CALM2_INSR		CM 8	CM 1	HSPG2	ITGB1	HSPG2_ITGB1
CM 8	CM 2	IGF1	INSR	IGF1_INSR		CM 8	CM 1	LAMA2	ITGB1	LAMA2_ITGB1
CM 8	CM 13	COL1A2	CD36	COL1A2_CD36		CM 8	CM 1	LGALS3BP	ITGB1	LGALS3BP_ITGB1
CM 8	CM 13	TIMP1	CD63	TIMP1_CD63		CM 8	CM 2	COL1A2	CD36	COL1A2_CD36
CM 8	CM 13	CALM2	INSR	CALM2_INSR		CM 8	CM 2	TIMP1	CD63	TIMP1_CD63
CM 8	CM 13	IGF1	INSR	IGF1_INSR		CM 8	CM 2	COL1A2	ITGB1	COL1A2_ITGB1
						CM 8	CM 2	COL3A1	ITGB1	COL3A1_ITGB1
						CM 8	CM 2	COL6A1	ITGB1	COL6A1_ITGB1
						CM 8	CM 2	COL6A2	ITGB1	COL6A2_ITGB1
						CM 8	CM 2	FN1	ITGB1	FN1_ITGB1
						CM 8	CM 2	HSPG2	ITGB1	HSPG2_ITGB1
						CM 8	CM 2	LAMA2	ITGB1	LAMA2_ITGB1
						CM 8	CM 2	LGALS3BP	ITGB1	LGALS3BP_ITGB1
						CM 8	CM 13	COL1A2	CD36	COL1A2_CD36
						CM 8	CM 13	TIMP1	CD63	TIMP1_CD63
						CM 8	CM 13	COL1A2	ITGB1	COL1A2_ITGB1
						CM 8	CM 13	COL3A1	ITGB1	COL3A1_ITGB1
						CM 8	CM 13	COL6A1	ITGB1	COL6A1_ITGB1
						CM 8	CM 13	COL6A2	ITGB1	COL6A2_ITGB1
						CM 8	CM 13	FN1	ITGB1	FN1_ITGB1
						CM 8	CM 13	HSPG2	ITGB1	HSPG2_ITGB1
						CM 8	CM 13	LAMA2	ITGB1	LAMA2_ITGB1
						CM 8	CM 13	LGALS3BP	ITGB1	LGALS3BP_ITGB1
